# Methanolic Extract of *Myrsine africana* Leaf Ameliorates Hyperglycemia and Dyslipidemia in Alloxan-Induced Diabetic Albino Mice

**DOI:** 10.1155/2021/3987656

**Published:** 2021-12-29

**Authors:** Yosef Eshetie Amare

**Affiliations:** Department of Biomedical Sciences, Institute of Medicine and Health Sciences, Debre Berhan University, Debre Berhan, Ethiopia

## Abstract

**Background:**

Diabetes mellitus is one of the leading public health problems globally, and its prevalence is increasing in Ethiopia. The current drugs for people with diabetes are costly, less effective, and less safe with a challenging administration method. Thus, globally, the need for alternative herbal antidiabetic medicines is increasing. In the previous studies, antioxidant activities have been seen in crude extracts of *M. africana* leaves, which is an auspicious sign of antidiabetic property. Accordingly, this study has evaluated the antidiabetic and antidyslipidemic activities of methanolic extract of *M. africana* leaves.

**Methods:**

Hypoglycemic and antihyperglycemic activities of the three doses (250 mg/kg, 500 mg/kg, and 1000 mg/kg) of crude methanolic extract of *M. africana* leaf were studied on normoglycemic, oral glucose-loaded, and alloxan-induced diabetic mice models. The effect of the extract on diabetic dyslipidemia, insulin and glycated hemoglobin levels, carbohydrate-metabolizing enzymes, and body weight was also studied in alloxan-induced diabetic mice. Glibenclamide (5 mg/kg) was used as a standard drug in all cases. Data analysis was carried out using mixed-design ANOVA. A *P* value of ≤0.05 was considered a statistically significant difference.

**Results:**

The methanolic extract of *M. africana* leaf did not show acute toxicity up to the dose of 5000 mg/kg and showed better glucose utilization in the oral glucose tolerance test. After 14 days of treatment, *M. africana* leaf extract decreased the blood glucose level, glycated hemoglobin, glucose-6-phosphatase, and fructose-1-6-bisphosphatase in diabetic mice. In contrast, it increased hexokinase and insulin levels in diabetic mice. Moreover, weight loss and dyslipidemia profiles have been corrected significantly in diabetic mice.

**Conclusion:**

*M. africana* leaves showed antihyperglycemic and antidyslipidemic effects in alloxan-induced diabetic mice. That suggests *M. africana* may be a potential treatment option for diabetes in the future. However, further molecular studies are required to analyze the mechanisms.

## 1. Introduction

Diabetes mellitus (DM) is a group of metabolic disorders characterized by increased blood glucose levels due to the absence of insulin secretion or defects in insulin action. It is becoming a worldwide public health problem leading to macro- and microvascular complications [1]. Moreover, hypercholesterolemia and hypertriglyceridemia are common complications of DM. In addition, elevated serum triglycerides, total cholesterol, and low serum high-density lipoprotein cholesterol levels exist in diabetic patients compared with nondiabetic individuals.

DM currently exerts a significant burden in the sub-Saharan region and is expected to increase. Many diabetic patients face significant challenges accessing diagnosis and treatment. This contributes to a high prevalence of mortality and complications [2]. Around 463 million people in the age group between 20 and 79 years have DM worldwide, which is predicted to increase to 700 million by 2045 [3].

Treatment of DM without side effects remained a challenge for the scientific community. As an example, the production of lactic acidosis, hypoglycemia, malabsorption, and weight gain or loss are shortcomings of contemporary antidiabetic medicines [4]. Therefore, exploring safer and more effective antidiabetic drugs is needed.

Globally, medicinal plants have been used as a source of modern medicine. About 80–85% of the world population relies on herbal extracts to treat diseases [5]. Historically, modern antidiabetic drugs were discovered from medicinal plants. One of the examples is *Galega officinalis*, that metformin was isolated from it [6].

*Myrsine africana* (*M. africana*), also called Cape Myrtle or African boxwood, belongs to the family *Myrsinaceae* and is an evergreen shrub growing to 2 m at a slow rate. The plant is native to Africa and Asia and usually grows well in dry parts [7].

Plant-derived secondary metabolites such as flavonoids, alkaloids, and phenolic compounds have a blood glucose-reducing ability [8–13]. Antihyperglycemic and antidyslipidemic effects of these plants are attributed to their ability to restore the function of pancreatic tissues by causing an increase in insulin production or inhibiting the intestinal absorption of glucose or the facilitation of metabolites in the insulin-dependent process. In hyperglycemic conditions, continuous generation of reactive oxygen species occurred. Reactive oxygen species increased oxidative stress mainly due to the overproduction of oxygen free radicals. The induction of oxidative stress is the leading cause of the advanced disease process of DM [8, 14, 15]. Antioxidants play an important role in hunting the free radicals, damaging the reactive oxygen species, and protecting the human body from oxidative stress. Therefore, scavenging of free radicals by antioxidants is one of the treatment and prevention strategies of DM [8, 15, 16].

The presence of saponins, flavonoids, tannins, and steroidal and phenolic compounds has been shown in the methanolic extract of *M. africana* leaf. Moreover, the result demonstrated that the extract of *M. africana* has intense antioxidant activity [17]. The *in vitro* study showed that *M. africana* has a vigorous antioxidant activity, which is a sign of promising antidiabetic activity [18, 19]. *M. africana* has shown a potential therapeutic effect for helminths, bacteria, and antitumor activities [20, 21]. However, the antidiabetic activity of *M. africana* is hitherto unknown. The antidiabetic and antidyslipidemic activities of *M. africana* leaf methanolic extract in alloxan-induced diabetic mice were evaluated in this study.

## 2. Materials and Methods

### 2.1. Drugs, Chemicals, and Instruments

This study used the following drugs, chemicals, and instruments: alloxan (Sigma Aldrich, Germany), methanol absolute (Nice Chemicals, India), glibenclamide (Julphar Pharmaceuticals, Ethiopia), normal saline (Addis Pharmaceutical Factory, Ethiopia), 40% glucose solution (Addis Pharmaceutical Factory, Ethiopia), PRODIGY^®^ blood glucose meter and strips (OK Biotech Co., Ltd., Taiwan), and Mindray BS-240 clinical chemistry analyzer (Shenzhen Mindray Bio-Medical Electronics Co., Ltd, China). All other chemicals used were of analytical grade.

### 2.2. Plant Material Collection and Extraction

The permission was obtained from Debre Berhan University institutional review board committee members (Dr. Esubalaew Tesfahun, Dr. Amare Ayalew, and Dr. Gezahegn Degafe). Fresh leaves of *M. africana* were collected from Ankober, North Shewa Zone of Amhara Region, Ethiopia, in May 2019. The use of plant parts in the present study complies with international, national, or institutional guidelines. The plant specimen was kept at the Herbarium of Addis Ababa University with the voucher number ATA0001 after taxonomic identification and authentication by Dr. Abiyou Tilahun. Then, dried fresh leaves (100 g) of the plant material were macerated, stayed for five days in stoppered conical flasks containing 200 ml of methanol, and filtered. The filtrate was dried under reduced pressure using a rotary evaporator to yield 3 g of crude extract. The weighed crude extract was stored at −4°C until used.

### 2.3. Experimental Animals

A total of 106 healthy Swiss albino mice of both sexes weighing 20–30 g were used in this study. This study was done following the ARRIVE guidelines. The animals were purchased from the Ethiopian Public Health Institute, Addis Ababa, Ethiopia. The animals were housed using standard cages and kept under normal conditions (12-hour light and 12-hours dark cycle) at the Department of Pharmacology, Addis Ababa University. The mice were acclimatized for seven days before the experiment and given a standard diet and water *ad libitum.*

### 2.4. Phytochemical Screening of *M. africana* Leaf Crude Extract

The absence or presence of terpenoids, steroids, flavonoids, glycosides, tannins, saponins, phenols, alkaloids, and anthraquinones was assessed in a crude extract of *M. africana* leaf using standard protocols.

Salkowski's test was applied to identify the presence of terpenoids. The methanolic extract (100 mg) of *M. africana* was dissolved in 5 ml of distilled water. The solution was mixed in 2 ml of chloroform and 3 ml of concentrated sulfuric acid. The formation of a reddish-brown color layer at the interface indicated the presence of terpenoids.

Braemer's test was applied for the detection of tannins. In a test tube, 0.25 g of the crude extract was stirred with 10 ml distilled water and filtered with filter paper (Whatman No. 1). Few drops of 2% ferric chloride were added to the filtrate. The filtrate was checked for green precipitation as an indication of the presence of tannins.

The Froth test was used to detect saponins. Five milliliters of distilled water and 0.25 g of the crude extract were added to a test tube. For 2 minutes, the solution was shaken robustly until a firm, persistent froth was seen. Froth formation indicates the existence of saponins.

The presence of anthraquinones was checked using Borntrager's test. 3 ml of the plant extract was dissolved in 3 ml of benzene and filtered with Whatman No. 1 filter paper. Then, 2 ml of 10% ammonium hydroxide was added. Purple ring formation will reveal anthraquinone's presence in the extract.

Detection of steroids was done using the Liebermann–Burchard test. The methanolic crude extract (0.5 g) was dissolved in 2 ml of distilled water in a test tube. Then, 2 ml of chloroform and sulfuric acid were added to the solution. The formation of red color at the lower layer of the chloroform layer indicated the presence of steroids.

Wagner's test was applied to detect the presence of alkaloids. Three drops of Wagner's reagent were added to 10 mg of the crude extract and dissolved in distilled water. Reddish-brown color formation reveals the presence of alkaloids.

The ferric chloride test was used to detect phenolic compounds. 0.5 ml of neutral 5% ferric chloride solution was added to the dissolved 10 mg crude extract with 1 ml distilled water. The blue-green color formation is indicative of the presence of phenolic compounds.

NaOH was used for the flavonoids test. Nearly 0.3 g of the crude extract was dissolved in 2 ml distilled water. Three drops of 20% NaOH solution were added to the solution. The presence of flavonoids is confirmed when the yellow color of the solution changed colorless after adding three drops of 20% hydrochloric acid.

Detection of glycosides was done using the Keller–Kiliani test. 20 ml of distilled water was used to dissolve 0.5 g of the dried crude extract in a test tube. The solution was filtered by Whatman No.1 filter paper after 24 hr. 5 ml of the filtrate was treated with 2 ml of concentrated glacial acetic acid and two drops of 0.1% ferric chloride solution. The mixture was poured into a test tube of 1 ml concentrated sulfuric acid. The brown ring formation at the interface is indicative of glycoside's presence in the extract [22–24].

### 2.5. Determination of Total Phenolic Content in *M. africana*

The Folin–Ciocalteu reagent was used to determine the total phenolic content. 200 *µ*g/ml of the extract was mixed with 400 *µ*l of the Folin–Ciocalteu reagent and 1.5 ml of 20% sodium carbonate. The mixture was shaken thoroughly and made up to 10 ml with distilled water. After 2 hours, the absorbance of the mixture was measured at 765 nm. The total concentration of phenol in *M. africana* extract was determined from the standard curve of gallic acid and was expressed as mg of gallic acid equivalent per gm of dried plant extract.

### 2.6. Determination of Total Flavonoid Content in *M. africana*

1 ml of the methanolic extract (200 *µ*g/ml) was mixed with 1 ml aluminum trichloride in 20 mg/ml methanol. A drop of acetic acid was added and then diluted with methanol to 20 ml. The solution was filtered through Whatman filter paper No. 1 before measuring the absorbance. Then, absorbance was measured at 415 nm against blank after 45 minutes. The total flavonoid content in *M. africana* was determined from the standard quercetin curve and was expressed as mg of quercetin equivalent per gm of dried plant extract.

### 2.7. Antioxidant Activity of *M. africana* Extract by DPPH Free Radical Scavenging Assay

The DPPH (2, 2 diphenyl-1-picrylhrazyl) free radical scavenging method was used to assess the antioxidant property of *M. africana.* Ascorbic acid was used as a control assuming that it has 100% free radical scavenging activity. 2.8 ml of DPPH solution (45 *µ*g/ml) was promptly added in 200 *µ*l of methanol solution of plant extract at different concentrations in test tubes. The solution was thoroughly mixed and kept for 30 minutes at room temperature. Finally, the absorbance was measured at 517 nm against methanol solution used as a blank. The percentage of scavenging of DPPH free radical is calculated using the following formula:

(1) $$\begin{array}{c} \left\{\frac{\left(A_{0}-A_{1}\right)}{A_{0}}\right\}\times 100, \end{array}$$

where *A*_0_ = absorbance of the control and *A*_1_ = absorbance of the extract/standard. Then, the percentage of inhibition was plotted against log concentration and IC_50_ was calculated from the graph.

### 2.8. Acute Oral Toxicity Study

An acute oral toxicity study of *M. africana* was performed based upon the organization for Economic Cooperation and Development (OECD) 425 guidelines [25]. Five female albino mice weighing 20–30 grams were used. After 4 hours fasting, a single mouse received 2000 mg/kg of the leaf extract orally and was left under careful observation for behavioral or/and physical changes for 24 hours. The remaining four female mice received the same dose, and strict follow-up continued until the end of fourteen days for potential signs of toxicity and mortality. The dose of the extract was increased to 5000 mg/kg, and a similar procedure was followed on another five female mice.

### 2.9. Induction of Diabetes

Alloxan (2,4,5,6-tetraoxypyrimidine; 5,6-dioxyuracil) was administered to induce diabetes. Before alloxan administration, the mice have been subjected to sixteen hours of fasting. Fresh alloxan solution was prepared using normal saline (0.9%) and administered intraperitoneally at the dose of 150 mg/kg according to previous studies [26–28]. The mice were screened for glucose levels after 72 hours of alloxan administration. Animals with fasting blood glucose levels >200 mg/dl were included in the study as diabetic mice [28–30]. After the screening, diabetic mice were randomly allocated to the different test groups.

### 2.10. Designs and Procedures of the Experiment

For the hypoglycemic, oral glucose tolerance, and single-dose treatment, the following testing groups were used, with six mice in each group: a negative control group (NS 10 ml/kg group or normal control), received 10 ml/kg NS; a positive control group (GLC 5 mg/kg group), treated with glibenclamide (5 mg/kg); and three MA-treated groups, received 250, 500, and 1000 mg/kg MA, which are MA 250 mg/kg group, MA 500 mg/kg group, and MA 1000 mg/kg group, respectively.

For the repeated daily dose treatment test, the following groups were used, with six mice in each group: a diabetic control mice group (diabetic control), received 10 ml/kg NS; a nondiabetic negative control group (normal control), received 10 ml/kg NS; a positive control mice group (GLC 5 mg/kg group), treated with glibenclamide (5 mg/kg); and three MA-treated groups, received 250, 500, and 1000 mg/kg MA, which are MA 250 mg/kg group, MA 500 mg/kg group, and MA 1000 mg/kg group, respectively.

Plant extract doses were determined based on acute oral toxicity results. The middle dose 500 mg/kg was one-tenth of the limit dose (5000 mg/kg), the higher dose 1000 mg/kg was twice the middle dose, and the lower dose 250 mg/kg was calculated as half of the middle dose The respective amount of the plant extract was administered orally to each animal after dissolving it with normal saline at a volume of not more than 10 ml/kg of body weight [31].

### 2.11. Determination of Blood Glucose Level

Blood samples were collected from the tail vein of each animal using an aseptic technique. According to the operating procedures, the blood glucose level was determined using the PRODIGY® blood glucose meter and strips (OK Biotech Co., Ltd., Taiwan). The test was done in triplicate.

For the hypoglycemic test, each group of mice received their respective treatment after overnight fasting. The BGL was measured at zero (just before treatment), one, two, four, and six hours after treatment [32, 33]. For the glucose tolerance test, glucose solution (40% w/v) was given orally to each animal at the dose of 2.5 g/kg after they got their respective treatment [32, 34]. Then, the BGL was measured at zero, 30, 60, and 120 minutes [32, 33]. For the single-dose antihyperglycemic test, overnight fasted diabetic mice received normal saline, *M. africana* leaf extract, and glibenclamide according to their groupings. Then, the BGL was recorded at zero (just before treatment), two, four, six, and eight hours [32, 33]. For the repeated daily dose antihyperglycemic test, mice were treated for 14 days based on their respective treatments. The BGL was recorded for each animal just before starting the treatment, on the first day of treatment (72 hours after alloxan administration) as a baseline, and on the seventh and fourteenth day. The mice were subjected to sixteen hours of fasting ahead of each sample collection [35].

### 2.12. Assessing the Body Weight and Serum Lipid Level Change on Repeated Daily Dose *M. africana*-Treated Diabetic Mice

The body weight of each animal was recorded just before the commencement of treatment, on the first day (72 hours after alloxan administration), and on the seventh and fourteenth day of respective therapy. After 14 days of treatment, the mice were euthanized using intraperitoneal (IP) injection of sodium pentobarbitone at a dose of 150 mg/kg [35, 36]. Then, the blood specimen was collected using a sterile gel tube via cardiac puncture. The blood samples were kept at room temperature for two hours and centrifuged. The serum lipid profiles, such as high-density lipoprotein, total cholesterol, and triglycerides, were measured using the Mindray BS-240 clinical chemistry analyzer (Shenzhen Mindray Bio-Medical Electronics Co., Ltd, China).

### 2.13. Biological Assays

The mice were anesthetized using sodium pentobarbitone. The blood sample of each mouse was collected using cardiac puncture and preserved with anticoagulant agents. The glycated hemoglobin was estimated according to the method of Nayak and Pattabiraman [37]. The insulin level was measured using an Insulin-1 ELISA kit (Sigma-Aldrich Germany). After 30 minutes, the color developed was measured at 450 nm. The test standard was done in duplicate.

A portion of the liver was dissected and washed out with saline immediately and was homogenized in 0.1 M HCl buffer (pH 7.4). The homogenate was centrifuged at 10 000 rpm to remove the debris, and the supernatant was used as an enzyme source for the assays of hexokinase [38], glucose-6-phosphatase [39], and fructose-1-6-bisphospahatase [40].

### 2.14. Data Management and Analysis

Data were recorded, summarized, and analyzed using SPSS, version 21, software (USA). The mean and standard deviation (SD) were calculated, and the association between and within groups was determined using mixed-design ANOVA. A *P* value of  ≤0.05 was considered indicative of a statistically significant difference.

## 3. Results

### 3.1. Phytochemical Screening

The phytochemical screening test of *M. africana* leaf crude extract showed the presence of terpenoids, steroids, flavonoids, glycosides, tannins, saponins, phenols, and alkaloids, as indicated in Table 1.

### 3.2. Quantitative Determination of Phenols and Flavonoids in *M. africana*

As indicated in Table 2, the total phenolic content was 40.34 ± 2.51 mg gallic acid equivalents per gram of *M. africana* extract, and the total flavonoid content was 36.28 ± 4.13 mg of quercetin equivalents per gram of *M. africana* extract.

### 3.3. Effect of *M. africana* on DPPH Free Radical Scavenging Activity

**Figure 1:**
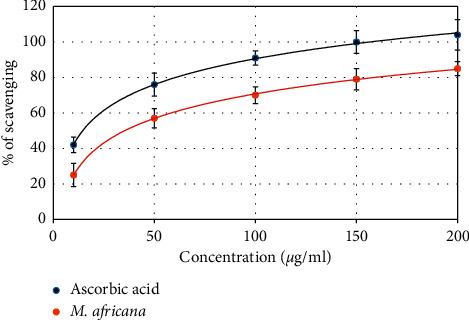
DPPH free scavenging (%) at various concentrations (µg/ml) of *M. africana* leaf methanolic extract and ascorbic acid. The antioxidant activity of the extract was estimated by the IC_50_ value.

The ability of *M. africana* on decreasing the production of DPPH free radicals is shown in Figure 1. Ascorbic acid, used as a standard, was extremely operative in inhibiting the DPPH free radicals, showing an IC50 of 13.21 *µ*g/ml, while the IC50 of M. africana was found to be 75.32 *µ*g/ml.

### 3.4. Acute Oral Toxicity Study

*M. africana* methanolic leaf extract did not cause mortality in mice up to 5000 mg/kg dose. None showed signs of toxicity (behavioral, neurological, or autonomic) in the first 24 hours up to 14 days of study duration. The LD_50_ of the *M. africana* leaf extract might be more than 5000 mg/kg.

### 3.5. Hypoglycemic Activity of *M. africana* Methanolic Leaf Extract in Normoglycemic Mice

**Figure 2:**
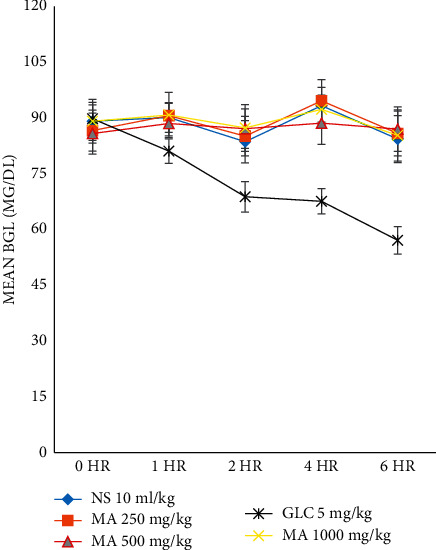
The hypoglycemic effects of *M. africana* leaf extract in normoglycemic mice. MA, *Myrsine africana* leaf extract; NS, normal saline; GLC, glibenclamide.

In all test groups, the baseline BGL was not different significantly when compared with each other (Table 3). At all time points, no statistical difference of BGL was seen between all the *M. africana*-treated groups and negative control groups (Figure 2); but the BGL of the glibenclamide (5 mg/kg)-treated group significantly decreased compared with that of the negative control group. Moreover, a statistically significant reduction of BGL was seen in the GLC-treated group when compared with all the *M. africana*-treated groups. At all time points, the BGL of all the *M. africana*-treated groups did not show a statistical difference when compared with each other. At all time points, there was no significant reduction of BGL in all three doses of the plant extract and normal saline-treated groups compared with the corresponding baseline level. However, the BGL in the GLC-treated group significantly decreased at the 1^st^ (*P* < 0.01), 2^nd^, 4^th^, and 6^th^ (*P* < 0.001) hours compared with the initial level.

### 3.6. Antihyperglycemic Activity of the Methanolic Leaf Extract of *M. africana* in Oral Glucose-Loaded Mice

**Figure 3:**
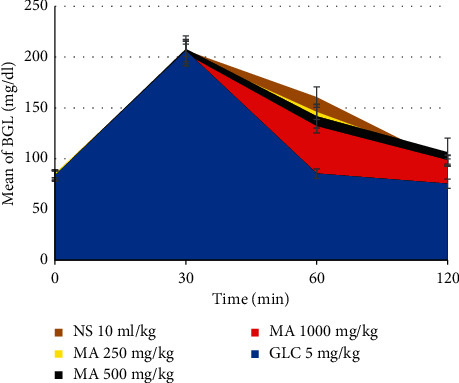
The area curve shows the blood glucose-lowering effect of *M. africana* leaf extract in the oral glucose tolerance test. MA, *Myrsine africana* leaf extract; NS, normal saline; GLC, glibenclamide.

As indicated in Table 4, the baseline BGL in all groups did not show a statistical difference when compared with each other. In all groups, a statistically significant (*P* < 0.001) increment of BGL was observed thirty minutes after oral glucose loading of each mouse (Figure 3). In all groups, after glucose loading, the BGL at the first and second hours has shown significant (*P* < 0.001) decrement when compared with the thirty-minute blood glucose level. In the first hour, MA 1000 mg/kg (*P* < 0.001) and MA 500 mg/kg (*P* < 0.01) have reduced hyperglycemia significantly when compared with the negative control. In the GLC-treated group, the state of hyperglycemia significantly reduced at the 1^st^ (*P* < 0.001) and 2^nd^ (*P* < 0.01) hours when compared with the normal saline-treated group. The BGL did not show significant differences in all the *M. africana*-treated groups when compared with each other. At the first and second hours, the BGL was significantly (*P* < 0.001) reduced in the GLC-treated group when compared with all the *M. africana*-treated groups.

### 3.7. Antihyperglycemic Activity of Single Dose of *M. africana* Methanolic Leaf Extract in Diabetic Mice

**Figure 4:**
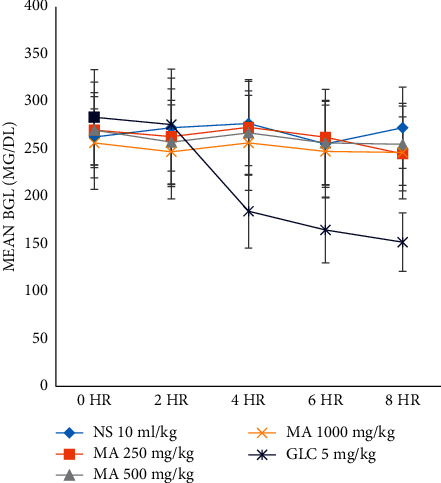
The blood glucose-lowering effect of *M. africana* leaf extract after single-dose administration in diabetic mice. MA, *Myrsine africana* leaf extract; NS, normal saline; GLC, glibenclamide.

At all time points, there was no significant BGL reduction seen in *M. africana*-treated groups when compared with the negative control group (Figure 4 and Table 5). However, the BGL was significantly decreased in the GLC-treated group at 4^th^ (*P* < 0.05), 6^th^ (*P* < 0.05), and 8^th^ (*P* < 0.001) hours when compared with the negative control group. Moreover, the BGL has shown substantial decrement at the second, fourth, and eighth hours in the GLC-treated group when compared with *M. africana*-treated groups. In the GLC-treated group, there was a significant reduction in the BGL at the 4^th^, 6^th^, and 8^th^ hours when compared with its baseline level.

### 3.8. Antihyperglycemic Activity of Repeated Daily Dose of *M. africana* Methanolic Leaf Extract in Diabetic Mice

**Figure 5:**
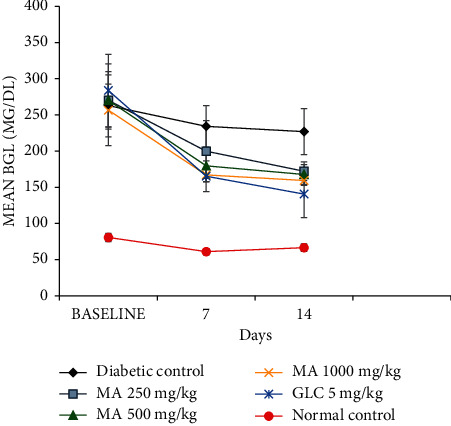
The blood glucose-lowering effect of *M. africana* leaf extract after repeated daily dose administration in diabetic mice. MA, *Myrsine africana* leaf extract; GLC, glibenclamide.

As indicated in Table 6, the baseline BGL was not significantly different in all diabetic mice groups when compared with each other. However, the BGL in the diabetic groups was significantly elevated (*P* < 0.001) when compared with the normal control group. On the 7^th^ day, the BGL has shown significant decrement (*P* < 0.05) in MA 500 mg/kg and MA 1000 mg/kg groups when compared with the diabetic control group. On the 14^th^ day, the result has revealed that a statistically significant reduction of BGL in all the *M. africana*-treated groups when compared with the diabetic control group (Figure 5). Besides, on the 7^th^ and 14^th^ days, a significant reduction of BGL was seen in GLC-treated group when compared with the diabetic control group. However, at all time points, the BGL was not statistically different between all *M. africana-*treated and GLC-treated groups. At all time points, the BGL of all the *M. africana*-treated groups did not show statistical differences when compared with each other. At the 7^th^ and 14^th^ days, the BGL has shown a significant reduction (*P* < 0.001) when compared with its baseline level in both GLC-treated and *M. africana*-treated groups.

### 3.9. Effect of Repeated Daily Dose of *M. africana* Methanolic Leaf Extract on Body Weight in Diabetic Mice

As indicated in Table 7, at the 7^th^ and 14^th^ days, the weight of animals in the diabetic control group has significantly dropped (*P* < 0.001) when compared with the normal control group. On the 7th day, there was a significant (*P* < 0.05) weight gain in the MA 1000 mg/kg group compared with the diabetic control group. In addition, on the 14^th^ day, a significant weight improvement was seen in all the *M. africana*-treated groups compared with the diabetic control group. At the 7^th^ and 14^th^ days, the weight of mice was significantly reduced in both MA 250 mg/kg and MA 500 mg/kg groups compared with the normal control group. In the GLC-treated group, a significant (*P* < 0.001) weight improvement was seen when compared with the diabetic control group at the 7^th^ and 14^th^ days.

### 3.10. Effect of Repeated Daily Dose of *M. africana* Methanolic Leaf Extract on the Serum Lipid Level in Diabetic Mice

**Figure 6:**
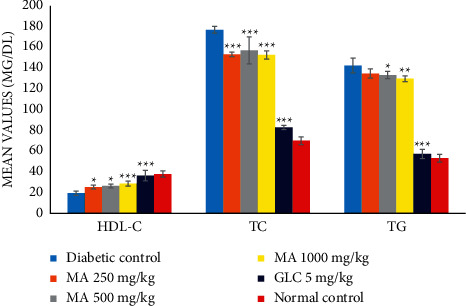
Trends of the effects of *M. africana* methanolic leaf extract on serum lipid profiles of diabetic mice. ^∗^*P* <0.05, ^∗∗^*P* <0.01, and ^∗∗∗^*P* <0.001 considered significant when compared with the diabetic control group. GLC, glibenclamide; MA, *Myrsine africana* leaf extract*;* HDL-C, high-density lipoprotein cholesterol; TC, total cholesterol; TG, triglyceride.

In the diabetic control group when compared with the normal control group, the levels of total cholesterol (TC) and triglycerides (TGs) increased significantly (*P* < 0.001); and high-density lipoprotein (HDL) cholesterol was significantly decreased (*P* < 0.001) (Table 8, Figure 6). After 14 days of treatment, when *M. africana*-treated groups compared with diabetic control, the TC and TG levels were decreased, while the HDL level increased significantly. Likewise, in comparing the GLC-treated group with diabetic control, a significant reduction of TG and TC and a significant increment of HDL were seen in the GLC-treated group (*P* < 0.001). The levels of TC, TG, and HDL-C did not show a statistically significant difference in all the *M. africana*-treated groups when compared with each other. In the GLC-treated group, the state of dyslipidemia was corrected better when compared with all the *M. africana*-treated groups.

### 3.11. Effect of *M. africana* on Insulin and Hexokinase

**Figure 7:**
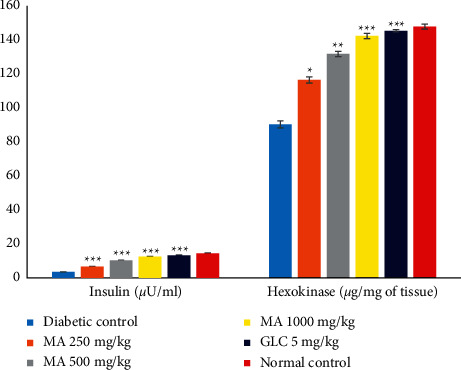
The effects of *M. africana* leaf extract on insulin and hexokinase levels in diabetic mice. ^∗^*P* <0.05, ^∗∗^*P* <0.01, and ^∗∗∗^*P* <0.001 considered significant when compared with the diabetic control group. MA, *Myrsine africana* leaf extract; GLC, glibenclamide.

The insulin level was significantly decreased in the diabetic control group compared with the normal control group, whereas the insulin level was significantly elevated (*P* < 0.001) in all the *M. africana*-treated groups compared with the diabetic control group. A more prominent increment of insulin level was seen in the MA 1000 mg/kg group when compared with other *M. africana*-treated groups (Figure 7, Table 9).

Likewise, the hexokinase level was significantly increased in all the *M. africana-*treated and GLC-treated groups compared with the diabetic control group. In *M. africana*-treated groups, the increment of hexokinase was dose dependent (Figure 7 and Table 9).

### 3.12. Effect of *M. africana* on Glycated Hemoglobin, Glucose-6-Phosphatase, and Fructose-1-6-Bisphosphatase

**Figure 8:**
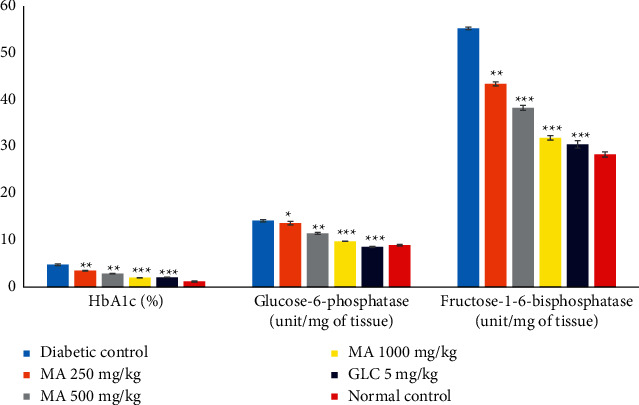
The effects of *M. africana* leaf extract on glycated hemoglobin, glucose-6-phosphatase, and fructose-1-6-bisphosphatase levels in diabetic mice. ^*∗*^*P* < 0.05, ^*∗∗*^*P* < 0.01, and ^*∗∗∗*^*P* < 0.001 considered significant when compared with the diabetic control group. HbA1c, glycated hemoglobin; MA, *Myrsine africana* leaf extract; GLC, glibenclamide.

The glycated hemoglobin was significantly increased in the diabetic control group. Conversely, a significantly decreased level of glycated hemoglobin was seen in all the *M. africana*-treated and GLC-treated groups when compared with the diabetic control group. The effect of *M. africana* on glycated hemoglobin was dose dependent (Figure 8 and Table 9).

The levels of glucose-6-phosphatase and fructose-1-6-bisphosphatase were significantly decreased in all the *M. africana*-treated and GLC-treated groups when compared with the diabetic control group (Figure 8 and Table 9).

## 4. Discussion

DM is the known metabolic disease described by elevated blood glucose levels due to compromised metabolism of macromolecules such as carbohydrates, lipids, and proteins and related to absolute or relative deficiencies in insulin secretion or/and insulin action [41]. The need for safe and efficient antidiabetic drugs is still the world scientific community issue. Exploring new modern medications from traditional medicinal plants is an important research area, which does not require advanced pharmaceutical settings and provides easier accessibility [9, 34].

The current study revealed that the methanolic extract from *M. africana* leaves can potentially treat diabetes mellitus. Acute oral toxicity test results of *M. africana* showed that the extract seems nontoxic (LD_50_ > 5000 mg/kg). The extract has a significant effect on decreasing blood glucose levels in diabetes-induced albino mice. The extract also showed the ability to increase the protective HDL level and reduce the unwanted lipid profiles. Moreover, *M. africana* has increased the insulin and hexokinase levels in diabetic mice, while it decreased the levels of glucose-6-phosphatase and fructose-1-6-bisphosphatase. Also, *M. africana* leaf extract might contribute to improved weight gains in diabetic mice.

Based on the results of this study, the LD_50_ of the methanolic leaf extract of *M. africana* is found to be more than 5000 mg/kg. In another study, the acute toxicity study of *M. africana* seed extract had shown the LD_50_ more than 5000 mg/kg [17].

Alloxan is a commonly used chemical for the induction of diabetes in rodents [42, 43]. This study has revealed the successful induction of diabetes in mice using alloxan compound. In another previous study as well, alloxan has induced persistent hyperglycemia in mice [26]. Likewise, in this study, sustained alloxan-induced hyperglycemia has been observed for a two-week study period as indicated in the diabetic control group (Table 6). After alloxan exposure, there will be pancreatic *β* cell malfunctioning due to DNA fragmentation and damage, production of superoxide radicals, and generation of reactive oxygen species [43].

The effect of the extract on normoglycemic animals suggests that the leaf of *M. africana* has a mild lowering effect on normal glucose levels (Table 3). This effect was comparable to that of glibenclamide, an insulin secretagogue, which also lowers blood glucose in normal animals. Provided the *β*-cells are fully functional, sulphonylureas, such as glibenclamide, can cause hypoglycemia since insulin release is initiated even when glucose concentration is below the normal threshold due to glucose-stimulated insulin release [32, 41, 44].

An oral glucose tolerance test is used to identify the altered carbohydrate metabolism during post-glucose administration. The ability of methanolic extract of *M. africana* leaf to lower the blood glucose level in oral glucose tolerance test suggests that mice treated with the doses of MA 1000 mg/kg and MA 500 mg/kg had better glucose utilization capacity. The reasons might be due to insulin emission from *β*-cells thereby that improved glucose transport and consumption [41].

The methanolic extract of *M. africana* leaf improved the blood glucose level in alloxan-induced diabetic mice. The *M. africana* dose 1000 mg/kg was most effective to reduce the raised blood glucose level (Table 6). The possible mechanism of activity of *M. africana* leaf extract may be stimulating the insulin secretion and regeneration of the *β*-cells of the pancreas or increasing cellularity of the islet tissue and regeneration of the granules in the *β*-cells. According to this result, it can be hypothesized that *M. africana* declined the level of blood glucose and improved the insulin level [26, 44], but further molecular studies are recommended to ascertain the actual mechanism for the antihyperglycemic effects noticed in this study.

There are strong experimental pieces of evidence that show that patients with diabetes mellitus are susceptible to an increase in the blood level of oxidants. In most medicinal plants, the presence of phytochemicals such as alkaloids, phenolic compounds, flavonoids, and terpenoids is responsible for their antidiabetic activity [8–13]. In recent years, much of the research interest was on the antioxidant activity of flavonoids due to their ability to reduce the formation and to scavenge the free radicals. Besides, the flavonoids have *β*-cell regenerating and insulinogenic effects [10, 11]. In this study also, the methanolic extract of *M. africana* has shown better antioxidant activity (Figure 1). Therefore, the presence of such phytonutrients in *M. africana* might contribute to the lowering of blood glucose levels [7, 17].

A diabetic state is characterized by severe loss in body weight because of loss or degradation of structural proteins. Due to insulin deficiency, there will be a marked reduction in the protein content in the muscular tissue via proteolysis [45]. This study also showed a significant body weight reduction in the diabetic mice group (Table 7), whereas the body weight was restored in *M. africana*-treated groups. The rebuilding of the weight loss in *M. africana*-treated groups may be due to cessation of proteolysis, gluconeogenesis, and glycogenolysis. Diabetes mellitus is associated with an increased rate of glycogenolysis, lipolysis, proteolysis, and gluconeogenesis [46]. The weight gain seen after repeated administration of *M. africana* might indicate its antihyperglycemic effect.

In the pathogenesis of diabetes, lipid plays a significant role. Increased levels of cholesterol and lipids in plasma represent a risk factor for coronary artery disease [46, 47]. Insulin deficiency causes activation of hormone-sensitive lipase that can lead to increased lipolysis and increased secretion of VLDL from the liver [47, 48]. Decreased activity of lipoprotein lipase, secondary to insulin deficiency, also leads to decreased clearance of chylomicrons and VLDL [49]. In addition, hypertriglyceridemia stimulates the enzymatic action of cholesterol ester transfer protein, which leads to an increase in the triglyceride content of LDL and HDL. Triglyceride-enriched HDL particles easily undergo catabolism, and triglyceride-enriched LDL particles undergo subsequent hydrolysis via hepatic lipase or lipoprotein lipase, resulting in reduced LDL particle size [47].

This study revealed a significant increment of serum TG and TC and a remarkable decrement of HDL-C in diabetic mice (Figure 6 and Table 8). Treating alloxan-induced diabetic groups with glibenclamide and different doses of *M. africana* leaf extract reduced the total cholesterol and triglyceride level to a significant extent in a dose-dependent manner, while it increased the beneficial HDL level to a substantial amount. The mechanism of the antidyslipidemic effect of *M. africana* is indirectly through controlled hyperglycemia or directly by influencing lipid metabolism.

In this study, the insulin level was decreased in the alloxan-induced diabetic mice. Different doses of *M. africana* leaf extract increased the insulin level, which may be due to active ingredients available in the plant extract. *M. africana* leaf extract may induce insulin secretion or maintain the function of *β*-cells from further damage, thereby keeping them active to produce insulin. Oral administration of the *M. africana* leaf extract for 14 days significantly decreased the blood glucose level with increasing insulin level. The mechanism of action of the plant extract might be by potentiating insulin secretion from *β*-cells, evidenced by this study that a significant elevation of insulin level was seen (Figure 7).

The glycated hemoglobin level was increased in alloxan-induced diabetic animals due to excessive glucose production in the blood, which further reacts with blood hemoglobin and prepared more glycated hemoglobin [50]. In this study, all doses of *M. africana* extract significantly lowered blood glucose and consequently decreased glycated hemoglobin levels. The possible mechanism of action might be due to having direct proportionality between blood glucose level and glycated hemoglobin. When the blood glucose level drops, the glycated hemoglobin will also decrease and vice versa.

The liver is a vital organ and plays an important role in defending the postprandial hyperglycemia and synthesis of glucose metabolism. The main role of the liver in glucose utilization is to convert the glucose into glucose-6-phosphate with the help of hexokinase, and another role is it converts glucose into energy [51, 52]. Alloxan-induced diabetic groups showed an increase in the level of glucose-6-phosphatase, which increased the production of fats to carbohydrates that deposited into the liver and kidneys and thus altered the level of hexokinase, which decreased the conversion and utilization of glucose. Another effect of diabetes is an increased level of fructose-1-6-bisphosphatase [52]. Treating alloxan-induced diabetic groups with different doses of M. africana leaf extract and glibenclamide increased the level of hexokinase and decreased the levels of glucose-6-phosphatase and fructose-1-6-bisphosphatase and brought the levels closer to the levels of normal control.

## 5. Conclusion

Thus, this study revealed that *M. africana* leaf extract has antidiabetic and antidyslipidemic effects, evidenced by decreased levels of blood glucose, glycated hemoglobin, glucose-6-phosphatase, fructose-1-6-bisphosphatase, total cholesterol, and triglyceride and increased levels of HDL-cholesterol, hexokinase, and insulin. The plant extract has also shown better glucose utilization in oral glucose tolerance tests and body weight improvements in diabetic mice. Further molecular studies are recommended to identify and understand the mechanisms of action of different compounds found in the plant.

## Figures and Tables

**Table 1:** Phytochemical screening of *M. africana* leaf crude extract.

List of metabolites	Tests used	Results
Terpenoids	Salkowski's test	+
Steroids	Liebermann–Burchard test	+
Flavonoids	NaOH test	+
Glycosides	Keller–Kiliani test	+
Anthraquinones	Borntrager's test	---
Tannins	Braemer's test	+
Saponins	Froth test	+
Phenols	Ferric chloride	+
Alkaloids	Wagner's test	+

**+**, indicates test positive; **---**, indicates test negative.

**Table 2:** Total phenolic and flavonoid content in *M. africana* leaf methanolic extract.

Parameters	Mean ± SD
Total phenolic content (mg of gallic acid equivalent/gm of dried plant extract)	40.34 ± 2.51
Total flavonoid content (mg of quercetin equivalent/gm of dried extract)	36.28 ± 4.13

**Table 3:** Hypoglycemic activity of the methanolic extract of *M. africana* leaf in normoglycemic mice.

Group	Fasting blood glucose level (mg/dl)				
	0 hr	1 hr	2 hr	4 hr	6 hr
NS 10 ml/kg	89.1 ± 5.1	90.1 ± 4	83.6 ± 5.7	93.3 ± 4.9	84.3 ± 6.3
MA 250 mg/kg	86.6 ± 3.3	90.6 ± 6.4	85.1 ± 6.3	94.6 ± 5.7	85.8 ± 6.4
MA 500 mg/kg	85.8 ± 5.5	88.5 ± 3.3	87.1 ± 5.3	88.6 ± 5.7	87 ± 6
MA 1000 mg/kg	89.1 ± 5.9	90.8 ± 6.1	87.3 ± 6.3	92.3 ± 3.8	85.3 ± 6.9
GLC 5 mg/kg	89.8 ± 3.7	81.1 ± 3.3^aE bE dE^^*βF*^	68.8 ± 4.1^aF bG cG dG^^*βG*^	67.6 ± 3.38^aG bG cG dG^^*βG*^	57.1 ± 3.7^aG bG cG dG^^*β*G^

Values are expressed as mean ± SD; *n* = 6; ^a^ compared with the negative control, ^b^ compared with MA 250 mg/kg, ^c^ compared with MA 500 mg/kg, ^d^ compared with MA 1000 mg/kg, and ^*β*^ compared with the baseline blood glucose level; ^E^*P* < 0.05, ^F^*P* < 0.01, ^G^*P* < 0.001; MA, *Myrsine africana* leaf extract; NS, normal saline; GLC, glibenclamide.

**Table 4:** Effect of *M. africana* methanolic leaf extract on oral glucose tolerance in normal mice.

Group	Fasting blood glucose level (mg/kg)			
	0 min	30 min	60 min	120 min
NS 10 ml/kg	84.8 ± 3.8	206.6 ± 10.8 ^*β*G^	160.6 ± 10.1 ^*π*G^	96.1 ± 3.6 ^*π*G^
MA 250 mg/kg	85.3 ± 3.9	204 ± 12.8 ^*β*G^	146 ± 7.6 ^*π*G^	98.8 ± 4.2 ^*π*G^
MA 500 mg/kg	83.3 ± 4.7	208 ± 7.5 ^*β*G^	141.6 ± 11.6 ^*π*G aF^	106.3 ± 14 ^*π*G^
MA 1000 mg/kg	83 ± 5.5	203.1 ± 9.6 ^*β*G^	132 ± 6.6 ^*π*G aG^	98.5 ± 5.2 ^*π*G^
GLC 5 mg/kg	83.5 ± 4.3	206.3 ± 14.7 ^*β*G^	85.3 ± 4.7 ^*π*G aG bG cG dG^	75.3 ± 4.6 ^*π*G aF bG cG dG^

Values are expressed as mean ± SD; *n* = 6; time refers to the time after oral glucose administration; ^a^ compared with the negative control, ^b^ compared with MA 250 mg/kg, ^c^ compared with MA 500 mg/kg, ^d^ compared with MA 1000 mg/kg, ^*β*^ compared with baseline blood glucose level, and ^*π*^ compared with the blood glucose level at 30 minutes; ^F^*P* < 0.01, ^G^*P* < 0.001; MA, *Myrsine africana* leaf extract; NS, normal saline; GLC, glibenclamide.

**Table 5:** Antihyperglycemic activity of a single dose of *M. africana* methanolic leaf extract in diabetic mice.

Group	Fasting blood glucose level (mg)				
	0 hr	2 hr	4 hr	6 hr	8 hr
NS 10 ml/kg	262.8 ± 29.7	272.5 ± 61.9	277 ± 44.4	255.5 ± 45.9	272.6 ± 42.8
MA 250 mg/kg	270.1 ± 50.5	263.1 ± 50.5	273 ± 50.3	262.6 ± 50.4	245 ± 39
MA 500 mg/kg	270 ± 39.6	257.3 ± 44	267.1 ± 44.2	256.5 ± 43.8	255.1 ± 43.2
MA 1000 mg/kg	256.5 ± 48.9	247.1 ± 49.4	256.6 ± 50.1	247.5 ± 48.9	246.5 ± 48.9
GLC 5 mg/kg	283.6 ± 50.1	275.8 ± 49	184.5 ± 38.8 ^*β*G aE bE cE^	164.8 ± 34.6 ^*β*G aE bF cE dE^	152 ± 30.8 ^*β*G aG bF cF dF^

Values are expressed as mean ± SD; *n* = 6; ^a^ compared with the negative control, ^b^ compared with MA 250 mg/kg, ^c^ compared with MA 500 mg/kg, ^d^ compared with MA 1000 mg/kg, and ^*β*^ compared with baseline blood glucose level; ^E^*P* < 0.05, ^F^*P* < 0.01, ^G^*P* < 0.001; MA, *Myrsine africana* leaf extract; NS, normal saline; GLC, glibenclamide.

**Table 6:** Antihyperglycemic activity of repeated daily dose of *M. africana* methanolic leaf extract in diabetic mice.

Group	Fasting blood glucose level (mg/dl)		
	Baseline	7^th^ day	14^th^ day
Diabetic control	262.8 ± 29.7 ^nG^	234.1 ± 28.8	226.8 ± 31.9
MA 250 mg/kg	270.1 ± 50.5 ^nG^	199.8 ± 42.3 ^*β*G^	172.1 ± 13 ^*β*G^^*α*F^
MA 500 mg/kg	270 ± 39.6 ^nG^	179.6 ± 17.4 ^*β*G^^*α*E^	167.6 ± 13.7 ^*β*G^^*α*G^
MA 1000 mg/kg	256.5 ± 48.9 ^nG^	167.1 ± 9.3 ^*β*G^^*α*E^	159.3 ± 6.9 ^*β*G^^*α*G^
GLC 5 mg/kg	283.6 ± 50.1 ^nG^	165.1 ± 21.3 ^*β*G^^*α*F^	140.5 ± 32.5 ^*β*G^^*α*G^
Normal control	80.6 ± 5.7	61 ± 4.3	66.5 ± 5.3

Values are expressed as mean ± SD; *n* = 6; ^*α*^ compared with diabetic control, ^*n*^ compared with normal control, ^*β*^ compared with baseline blood glucose level; ^E^*P* < 0.05, ^F^*P* < 0.01, ^G^*P* < 0.001; MA, *Myrsine africana* leaf extract; NS, normal saline; GLC, glibenclamide.

**Table 7:** Effect of the repeated daily dose of *M. africana* methanolic leaf extract on body weight in diabetic mice.

Group	Body weight (gm.)			
	Before diabetes induction	Baseline	7^th^ day of treatment	14^th^ day of treatment
Diabetic control	24.3 ± 4.1	19.3 ± 4	19.6 ± 3.2 ^nG^	15 ± 2.8 ^*β*F nG^
MA 250 mg/kg	23 ± 3.2	18.3 ± 1.8	23 ± 1.2 ^nE^	19.6 ± 2.4 ^nG^^*α*E^
MA 500 mg/kg	25 ± 1.5	19.3 ± 3	21.6 ± 2.4 ^nF^	22.3 ± 2.5 ^nE^^*α*G^
MA 1000 mg/kg	23 ± 3.2	18.3 ± 2.8	24.3 ± 2.5 ^*α*E^	22.6 ± 2 ^*α*G^
GLC 5 mg/kg	25 ± 3.5	19.3 ± 2	27.6 ± 1.8 ^*α*G^	26.8 ± 1.4 ^*α*G^
Normal control	23 ± 2.2	21.6 ± 2.1	28 ± 1.8	26.8 ± 2.1

Values are expressed as mean ± SD; *n* = 6; ^*α*^ compared with diabetic control, ^*n*^ compared with normal control, ^*β*^ compared with baseline blood glucose level; ^E^*P* < 0.05, ^F^*P* < 0.011, ^G^*P* < 0.001; MA, *Myrsine africana* leaf extract; NS, normal saline; GLC, glibenclamide.

**Table 8:** Effect of the repeated daily dose of *M. africana* methanolic leaf extract on the serum lipid level in diabetic mice.

Group	Serum lipid level (mg/dl)		
	HDL-C	TC	TG
Diabetic control	19.6 ± 2.1 ^nG^	177.3 ± 3.3 ^nG^	142.8 ± 7.1 ^nG^
MA 250 mg/kg	25.5 ± 1.8 ^nG^^*α*E^	153.6 ± 2.1 ^nG^^*α*G^	135.1 ± 4.4 ^nG^
MA 500 mg/kg	26.5 ± 1.8 ^nG^^*α*E^	157.5 ± 13 ^nG^^*α*G^	133.6 ± 3.5 ^nG^^*α*E^
MA 1000 mg/kg	28.8 ± 2.4 ^nG^^*α*G^	153 ± 4 ^nG^^*α*G^	130.1 ± 2.8 ^nG^^*α*F^
GLC 5 mg/kg	36.6 ± 5 ^*α*G bF cF dF^	83 ± 2 ^*α*G bF cF dF^	57.5 ± 4.5 ^*α*G bF cF dF^
Normal control	38.1 ± 3.1	70.3 ± 3.7	53.3 ± 3.7

Values are expressed as mean ± SD; *n* = 6; ^*α*^ compared with diabetic control, ^b^ compared with MA 250 mg/kg, ^c^ compared with MA 500 mg/kg, ^d^ compared with MA 1000 mg/kg, and ^n^ compared with normal control; ^E^*P* < 0.05, ^F^*P* < 0.01, ^G^*P* < 0.001; MA, *Myrsine africana* leaf extract; GLC, glibenclamide; TC, total cholesterol; TG, triglyceride; HDL-C, high-density lipoprotein cholesterol.

**Table 9:** Effect of *M. africana* methanolic leaf extract on glycated hemoglobin, insulin, and carbohydrate metabolizing enzymes in diabetic mice.

Groups	Biochemical parameters				
	HbA1c (%)	Insulin (*µ*U/ml)	Hexokinase (*µ*g/mg of tissue)	Glucose-6-phosphatase (unit/mg of tissue)	Fructose-1-6-bisphosphatase (unit/mg of tissue)
Diabetic control	4.8 ± 0.18 ^***π***^^*∗∗∗*^	3.6 ± 0.06 ^***π***^^*∗∗∗*^	90.58 ± 2.06 ^***π***^^*∗∗∗*^	14.2 ± 0.23 ^***π***^^*∗∗∗*^	55.38 ± 0.27 ^***π***^^*∗∗∗*^
MA 250 mg/kg	3.5 ± 0.09 ^***α***^^*∗∗*^	6.8 ± 0.05 ^*α*^^*∗∗∗*^	116.82 ± 1.85 ^***α***^^*∗*^	13.7 ± 0.36 ^***α***^^*∗*^	43.5 ± 0.43 ^***α***^^*∗∗*^
MA 500 mg/kg	2.9 ± 0.05 ^***α***^^*∗∗*^	10.4 ± 0.17 ^***α***^^*∗∗∗*^	132.12 ± 1.52 ^***α***^^*∗∗*^	11.5 ± 0.19 ^***α***^^*∗∗*^	38.34 ± 0.52 ^***α***^^*∗∗∗*^
MA 1000 mg/kg	2 ± 0.02 ^***α***^^*∗∗∗*^	12.6 ± 0.09 ^***α***^^*∗∗∗*^	142.73 ± 1.54 ^***α***^^*∗∗∗*^	9.8 ± 0.03 ^***α***^^*∗∗∗*^	31.94 ± 0.48 ^***α***^^*∗∗∗*^
GLC 5 mg/kg	2.1 ± 0.06 ^***α***^^*∗∗∗*^	13.4 ± 0.27 ^***α***^^*∗∗∗*^	145.79 ± 0.63 ^***α***^^*∗∗∗*^	8.6 ± 0.14 ^***α***^^*∗∗∗*^	30.55 ± 0.8 ^***α***^^*∗∗∗*^
Normal control	1.2 ± 0.15	14.6 ± 0.25	148.25 ± 1.51	9 ± 0.15	28.39 ± 0.55

Values are expressed as mean ± SD; *n* = 6; ^***π***^ compared with normal control; ^***α***^ compared with diabetic control; ^∗^*P* <0.05, ^∗∗^*P* <0.01, ^∗∗∗^*P* <0.001; HbA1c, glycated hemoglobin; MA, *Myrsine africana* leaf extract.

## Data Availability

The data used to support the findings of this study are available from the corresponding author upon request.

## Abbreviations

ANOVA: Analysis of variance
BGL: Blood glucose level
DM: Diabetes mellitus
GLC: Glibenclamide
HDL: High-density lipoprotein
HDL-C: High-density lipoprotein cholesterol
IDF: International Diabetes Federation
IP: Intraperitoneal
LD_50_: Median lethal dose
NS: Normal saline
OECD: Organization for Economic Cooperation and Development
MA: *Myrsine africana*
SD: Standard deviation
SPSS: Statistical Software Package for Social Science
TC: Total cholesterol
TG: Triglyceride
VLDL: Very low-density lipoprotein.
